# Molecular Basis of Efficient Replication and Pathogenicity of H9N2 Avian Influenza Viruses in Mice

**DOI:** 10.1371/journal.pone.0040118

**Published:** 2012-06-29

**Authors:** Xiaokang Li, Wenbao Qi, Jun He, Zhangyong Ning, Yue Hu, Jin Tian, Peirong Jiao, Chenggang Xu, Jianxin Chen, Juergen Richt, Wenjun Ma, Ming Liao

**Affiliations:** 1 College of Veterinary Medicine, South China Agricultural University, Guangzhou, China; 2 Department of Diagnostic Medicine/Pathobiology, Kansas State University, Manhattan, Kansas, United States of America; 3 College of Animal Science and Technology, Henan University of Science and Technology, Luoyang, China; University of Georgia, United States of America

## Abstract

H9N2 subtype avian influenza viruses (AIVs) have shown expanded host range and can infect mammals, such as humans and swine. To date the mechanisms of mammalian adaptation and interspecies transmission of H9N2 AIVs remain poorly understood. To explore the molecular basis determining mammalian adaptation of H9N2 AIVs, we compared two avian field H9N2 isolates in a mouse model: one (A/chicken/Guangdong/TS/2004, TS) is nonpathogenic, another one (A/chicken/Guangdong/V/2008, V) is lethal with efficient replication in mouse brains. In order to determine the basis of the differences in pathogenicity and brain tropism between these two viruses, recombinants with a single gene from the TS (or V) virus in the background of the V (or TS) virus were generated using reverse genetics and evaluated in a mouse model. The results showed that the PB2 gene is the major factor determining the virulence in the mouse model although other genes also have variable impacts on virus replication and pathogenicity. Further studies using PB2 chimeric viruses and mutated viruses with a single amino acid substitution at position 627 [glutamic acid (E) to lysine, (K)] in PB2 revealed that PB2 627K is critical for pathogenicity and viral replication of H9N2 viruses in mouse brains. All together, these results indicate that the PB2 gene and especially position 627 determine virus replication and pathogenicity in mice. This study provides insights into the molecular basis of mammalian adaptation and interspecies transmission of H9N2 AIVs.

## Introduction

Since the first H9N2 subtype avian influenza virus (AIV) was isolated from turkeys in 1966 in the U.S. [1], this subtype of viruses has been circulating in birds worldwide. Although H9N2 viruses are often found in shorebirds and wild ducks in North America [2], there is no evidence of a permanent lineage of these viruses in land-based poultry [3]. In Asia, H9N2 subtype of AIVs has become endemic in domestic poultry in many countries [4], [5], [6], [7], [8], [9]. Noticably, H9N2 viruses have transmitted from land-based chickens to pigs [10], [11], [12], [13], [14], [15]. Also, H9N2 viruses have been reported to infect humans and caused mild respiratory disease [12], [16], [17]. Further evidence of its mammalian host range is that some H9N2 strains replicate efficiently in mice and are able to kill mice without prior adaptation [5], [18]. All these facts indicate that H9N2 AIVs have expanded their host range and are able to infect different mammalian hosts including humans.

Although infections of humans with H5, H7 and H9 AIVs have been documented, the molecular mechanism for adaptation of AIVs in mammalian hosts remains poorly understood. The surface protein hemagglutinin (HA), which is responsible for binding of the virus to cellular receptors, is a major determinant in the host range of influenza A viruses. The HAs of AIVs preferentially bind to α2,3 sialic acid receptors, whereas the HAs from human influenza viruses preferentially bind to α2,6 sialic acid receptors [19]. Normally, the receptor binding site of the H9N2 HA similar to other AIVs’ HAs contains 226Q (glutamine, Q) and 228G (Glycine, G); an increasing number of currently circulating H9N2 isolates carry an leucine (L) at position 226 in the receptor binding site, a position which has been shown to be critical for influenza replication efficacy in human airway epithelial cells [20]. The polymerase PB2 gene is also a major factor of host range for human influenza viruses and highly pathogenic H5N1 AIVs; a single-amino-acid substitution at position 627 of the PB2 protein from glutamic acid (E) to lysine (K) is responsible for virulence in mammalian species [21], [22], [23]. Previous adaption studies showed that mouse adapted H9N2 viruses contain PB2 627K which is associated with efficient replication and virulence of H9N2 AIV in mice [3], [24].

In this study, we characterized two H9N2 AIVs that were isolated from chickens, A/chicken/Guangdong/TS/2004 (TS) and A/chicken/Guangdong/V/2008 (V) in southeastern China. These two viruses showed similar pathogenicity for chickens, but differ significantly in virulence and tissue tropism in mice. To determine the molecular basis for the difference in virulence and tissue tropism in mice, we generated recombinant and mutated viruses via reverse genetics and tested them in the mouse model. Our results showed that the single amino acid substitution at position 627 of the PB2 protein from E to K contributes to efficient replication and lethality of H9N2 AIVs in mice.

## Materials and Methods

### Cells and Viruses

Human embryonic kidney cells (293T) were purchased from the China Center for Type Culture Collection (CCTCC) and maintained in Dulbecco’s modified Eagle’s medium (DMEM) supplemented with 10% fetal bovine serum and 1% antibiotics, and were incubated at 37°C in 5% CO_2_. The H9N2 influenza A virus A/chicken/Guangdong/TS/2004 (TS) was isolated from the lung of a dead chicken in a poultry farm with an outbreak of respiratory disease in Guangdong, China, in 2004. The H9N2 influenza A virus A/chicken/Guangdong/V/2008 (V) was isolated from rectal swabs of diseased chickens in Guangdong, China, in 2008; the diseased chickens showed sneezing, depression and diarrhea with a low mortality. Both viruses were amplified in 10-day-old SPF embryonated eggs and used in this study.

### Construction of Plasmids

To establish eight-plasmid reverse genetic systems for the TS and V viruses, a bidirectional transcription vector (pDL) was used. The pDL contains human RNA pol I promoter and murine RNA polymerase I terminator sequences, which are flanked by the RNA polymerase II promoter of human cytomegalovirus and SV40 late polyadenylation signal. Two BsmB I restriction sites were utilized to clone viral full-length cDNA between RNA pol I promoter and terminator. The viral cDNAs were amplified by RT-PCR with primers containing *Bsm*B I sites (primers are available upon request), and then digested with *Bsm*BI, and cloned into the *Bsm*BI sites of the pDL vector. The resulting plasmids (pDL-V-PB2, -PB1, -PA, -HA, -NP, -NA, -M and –NS; pDL-TS-PB2, -PB1, -PA, -HA, -NP, -NA, -M and –NS) were confirmed by sequencing (primers are available upon request). Mutations were introduced into the PB2 gene by site-directed mutagenesis Kit (Invitrogen). The resulting plasmids are pDL-V-PB2-627E and pDL-TS-PB2-627K, which were confirmed by sequencing. The plasmids for transfection were prepared by using the Perfectprep Plasmid mini kit (Eppendorf, Hamburg, Germany).

### Generation of Recombinant Viruses Using Reverse Genetics

An eight plasmid reverse genetic system was used to generate wild-type and recombinant viruses. A monolayer of 293T cells with approximately 90% confluence in six-well plates was transfected with 5 µg of the eight plasmids (0.6 µg/each plasmid) by using Lipofectamine 2000 (Invitrogen) according to the manufacturer’s instructions. Briefly, 5 µg of plasmids and 10 µL of lipofectamine 2000 were mixed, incubated at room temperature for 30 min, and then added to the cells. After 6 hours incubation at 37°C, the mixture was replaced with DMEM containing 2% fetal bovine serum and 0.2 µg/mL TPCK-treated trypsin. The supernatant was harvested after 2 days incubation and 100 µL of supernatant was injected into an embryonated egg for virus propagation. The inoculated eggs were incubated for 3 days and the allantoic supernatant was collected and tested by hemagglutination assay. The rescued viruses were confirmed by sequencing of the whole viral genome.

### Chicken Experiment

To determine the pathogenicity of the two H9N2 viruses, the Intravenous Pathogenicity Index (IVPI) test was performed according to the recommendation of the Office International Des Epizooties (OIE). The chicken experiment was approved by the Institutional Animal Care and Use Committee at South China Agricultural University. Twenty-four 6-week-old SPF white leghorn chickens were randomly allocated into 3 groups (8 chickens/group). Each chicken was intravenously inoculated through the wing vein with 0.2 mL of inoculum containing 1∶10 of virus (original virus HA titer ≥1024) or 0.2 mL of sterile phosphate-buffered saline (PBS) in the control group. The birds were monitored daily for 10 days and scored based on the OIE recommended scoring system: 0 if normal, 1 if sick, 2 if very sick and 3 if dead.

### Mouse Experiments

Four-week-old female BALB/c mice (Guangdong Experimental Animal Center, Guanzhou, China) were used in this study. All mouse experiments were approved by the Institutional Animal Care and Use Committee at South China Agricultural University. Mice were intranasally inoculated with 10^6^ 50% egg infection doses (EID_50_) of each virus per mouse in 40 µL sterile PBS or 40 µL sterile PBS in the control group under light anesthesia. Mice were monitored daily for weight loss and clinical signs. If a mouse lost body weight over 25% of its pre-infection weight, it was defined as dead and humanely euthanized immediately; the rest of the mice were sacrificed at the end of experiment on 10 days post infection (dpi). The 10% of homogenate of each organ tissue in PBS was used for virus titration in 10-day-old embryonated chicken eggs. Virus titers were given in units of log_10_EID_50_ per mL ± standard deviation (SD).

In the first experiment, 90 mice were randomly divided into 3 groups (30 mice/group). Mice were intranasally infected with the TS or V virus, or PBS as controls. Three mice in each group were euthanized to investigate viral replication and tissue tropism from 1 to 10 dpi. The tissues collected from each animal included heart, liver, spleen, lung, kidney, duodenum, rectum and brain. Histopathological analysis were performed using H & E and IHC staining (using an in-house anti-HA monoclonal antibody as the first antibody) on samples collected on 5 dpi.

In subsequent experiments, 11 mice were used in each group. Three mice in each group were euthanized on 3 and 5 dpi. The remaining 5 mice were monitored daily for weight loss and mortality until 10 dpi. During necropsy, lung and brain were collected for virus titration.

### Statistical Analyses

The log_10_ transformed virus titers were analyzed using ANOVA, with a *P*-value ≤0.05 considered statistically significant (GraphPad Prism, GraphPad Software, La Jolla, CA). Virological measures shown to be significantly different by treatment group were compared pairwise by using the Tukey–Kramer test.

## Results

### Virulence of TS and V H9N2 Viruses in Chickens and Mice

**Figure 1:**
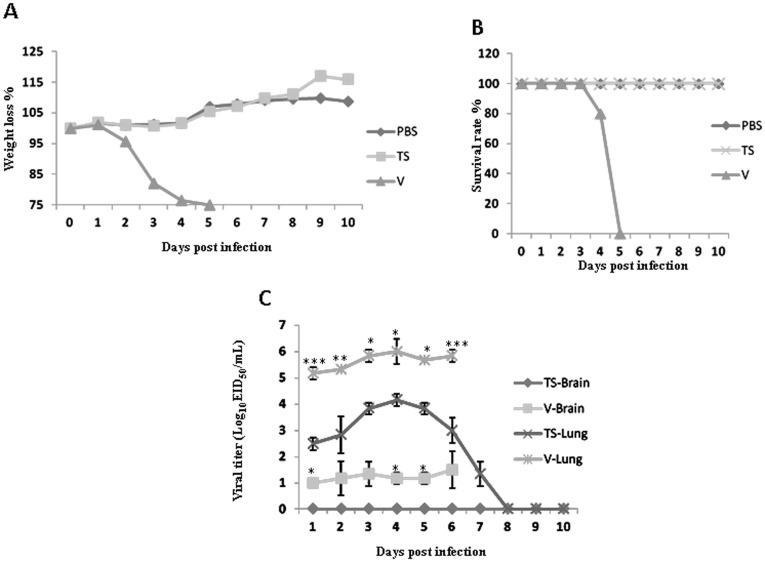
Weight changes, mortality and virus titers of mice inoculated with the TS and V virus. A) Weight changes of mice; B) Mortality of mice; C) Virus titers of the mouse lungs and brains: four-week-old SPF BALB/c mice (thirty mice/group) were inoculated intranasally with 10^6^ EID_50_ of each virus and three mice were euthanized on each day post infection and organ tissues were collected for virus titration in eggs. Data shown are the log_10_ geometric mean EID_50_/ml ± SEM; dashed line indicates detection limit of 10^1^ EID_50_/ml.

Chickens inoculated with either TS or V virus did not show any clinical signs during the experimental period, similar to the control chickens. The IVPI of both TS and V H9N2 viruses is 0, indicating they are nonpathogenic for chickens although they were isolated from diseased or dead chickens. In infected mice, the TS virus did not induce weight loss and any clinical signs, whereas the V virus caused severe weight loss and clinical signs including decreased activity, huddling, hunched posture and ruffled fur (**Figure 1A**). The V virus caused 100% mortality in infected mice, whereas no mice died in the TS virus infected group (**Figure 1B**). Both viruses were able to replicate in mouse lungs; however, the V virus grew to significant higher titers than the TS virus. Interestingly, besides the lung the V virus was also detected in brains of infected mice, but not in other tissues. In contrast, the TS virus was not found in the brain and other tissues with the exception of the lungs (**Figure 1C**). Both the V and TS viruses induced moderate bronchopneumonia in infected mice. The TS caused slightly fewer microscopic lung lesions in infected mice on 5 dpi than did the V virus; no significant difference was observed among the groups inoculated with the V or TS virus (data not shown). The V virus induced nonsuppurative encephalitis with proliferation of neuroglial cells in infected mice on 5 dpi, whereas the TS virus did not. This finding was confirmed by detecting viral antigens in the brains of mice infected with the V but not the TS virus using IHC staining (data not shown).

### Virulence of Wild-type and Recombinant Viruses in Mice

**Figure 2:**
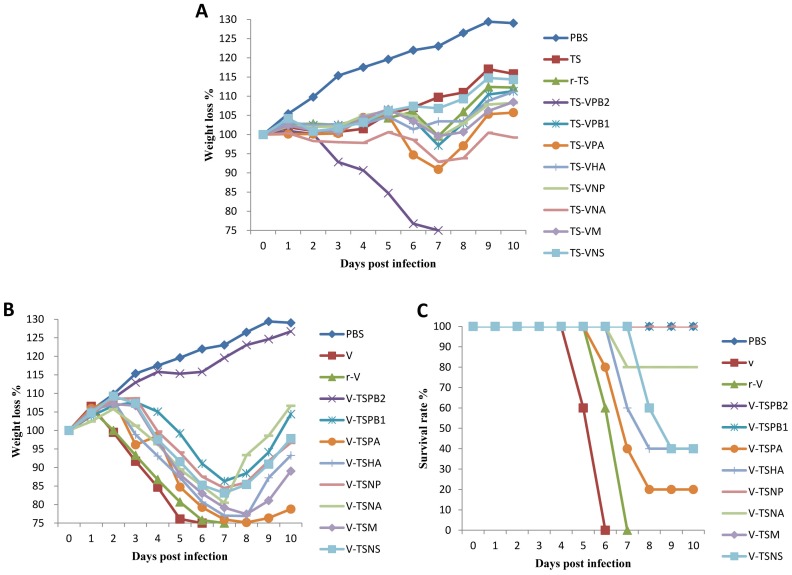
Weight changes of mice infected with wild-type, parental and recombinants viruses. A) Weight changes of mice inoculated with the wild-type TS, parental r-TS and recombinant viruses. B) Weight changes of mice inoculated with the wild-type V, parental r-V and recombinant viruses.

The eight plasmid reverse genetic system was established for both the TS and V viruses. Both r-TS and r-V viruses generated by reverse genetics retained the biological properties of their wild-type viruses in terms of pathogenicity and tissue tropism in mice as described above (**Table 1** **,** **Figure 2**).

**Table 1:** Virus replication in mouse lungs and brains on 3 and 5 days post infection.

Viruses	Lung		Brain	
	3 dpi	5 dpi	3 dpi	5 dpi
TS	3.83±0.14^a^	3.75±0.14	<1^b^	<1
r-TS	3.91±0.05	3.97±0.05	<1	<1
TS-VPB2	5.67±0.38	5.67±0.38	1.11±0.14	1.0±0.43
TS-VPB1	4.00±0.29	4.67±0.38	<1	<1
TS-VPA	4.33±0.38	4.33±0.50	<1	<1
TS-VHA	3.91±0.43	4.33±0.43	<1	<1
TS-VNP	4.67±0.50	4.67±0.43	<1	<1
TS-VNA	4.67±0.43	4.67±0.43	<1	<1
TS-VM	4.11±0.29	4.91±0.29	<1	<1
TS-VNS	3.11±0.14	3.11±0.29	<1	<1
V	5.97±0.06	6.03±0.06	1.42±0.53	1.35±0.32
r-V	6.01±0.05	6.07±0.04	1.33±0.66	1.25±0.66
V-TSPB2	4.91±0.38	4.91±0.43	<1	<1
V-TSPB1	5.00±0.43	5.33±0.29	<1	<1
V-TSPA	6.00±0.43	5.91±0.14	<1	<1
V-TSHA	6.11±0.43	5.33±0.50	<1	<1
V-TSNP	5.33±0.14	5.67±0.43	<1	<1
V-TSNA	5.67±0.29	5.67±0.43	<1	<1
V-TSM	5.33±0.29	5.67±0.38	<1	<1
V-TSNS	6.00±0.38	5.67±0.29	<1	<1

a Numbers are log_10_ geometric mean EID_50_/ml ± SEM.

b The detection limit is 10^1^ EID_50_/ml.

To determine the genes responsible for differences in virulence between the TS and V viruses in mice, eight recombinant viruses containing a single gene from the V virus in the genetic background of the TS virus (designated TS-VPB2, TS-VPB1, TS-VPA, TS-VHA, TS-VNA, TS-VNP, TS-VM and TS-VNS) were generated and their replication and virulence was tested in mice (**Table 1**). Several recombinant viruses including TS-VPA and TS-VNA caused transient weight loss (approximately 10% at 7 dpi) and clinical signs in infected mice, but only the TS-VPB2 caused severe disease with 100% mortality (**Figure 2A**). The recombinant TS-VPB2 virus grew to a significant higher virus titer in mouse lungs than the parental r-TS virus; no significant differences in lung virus titers were observed among the other 7 recombinant and the parental viruses. Interestingly, only the TS-VPB2 (3 and 5 dpi) virus was also found in mouse brains similar to the parental V virus (**Table 1**). These results indicated that PB2 from the V virus is critical for virus replication and virulence in mice.

In addition, eight recombinant viruses containing a single gene from the TS virus in the genetic background of the V virus (designated V-TSPB2, V-TSPB1, V-TSPA, V-TSHA, V-TSNA, V-TSNP, V-TSM and V-TSNS) were generated and tested in mice. When compared to the parental r-V virus, the recombinant viruses except V-TSPB2 caused obvious weight loss and clinical signs in infected mice (**Figure 2B** **)**; the parental r-V was attenuated due to the introduction of a single gene from the TS virus although recombinant V-TSPA, V-TSHA, V-TSNS and V-TSNA viruses still induced mortality in infected mice **(** **Figure 2C** **)**. Mice infected with the recombinant V-TSPB2 virus exhibited no weight loss and no clinical signs and they gained weight similar to the control mice (**Figure 2B**). All recombinant viruses were able to replicate in mouse lungs, but the recombinant viruses containing PB2, PB1, NP and M from TS virus replicated to lower titers when compared to the parental r-V and other recombinant viruses (**Table 1**). Noticeably, no virus was found in mouse brains infected with any of recombinant viruses. These results indicated that genes from the TS virus affected viral replication and tissue tropism of recombinant viruses in the genetic background of the V virus. All above results indicated that PB2 plays a major role for the observed differences in viral replication, tissue tropism and pathogenicity in mice.

### Sequence Comparison of TS and V Viruses

The full genome sequence analysis showed both viruses had 90.7–98.2% identity at the nucleotide level and 93.8–100% identity at the amino acid level between genes/proteins of the TS and V viruses (GenBank accession No.: JQ639775–JQ639790). There were a total of 101 amino acid differences between these two viruses. In the surface proteins, there were 15 amino acid differences in the HA protein and 29 amino acid differences in the NA protein between the TS and V viruses (**Table 2**). Noticeably, both HAs contain 226Q that has been demonstrated to bind avian-like influenza receptors [19] and there was a 3 (62–64) amino acid deletion in the stalk of the NA of the TS virus (**Table 2**). In the polymerase proteins, a difference of 10 amino acids was observed in both the PB1 and PB2 proteins; whereas there were 8 amino acid differences in the PA protein (**Table 3**). The amino acids at positions 613 (V) and 627 (E) in the PB2 of the TS were avian-like [25], whereas human-like influenza signatures were found at the respective positions (613I and 627K) in the V PB2 (**Table 3**). The TS virus expresses a full-length (90 amino acids) PB1-F2 protein, whereas the V virus expresses a truncated PB1-F2 (79 amino acids) since there is a stop codon (amino acid position 80) in the V PB1-F2 open reading frame. In the NP protein one human-like signature at position 214 (K) was found in the V virus, whereas at the same position an avian-like signature (214R) is presented in the TS virus. There were various amino acid differences in the M2, NS2 and the nonstructural NS1 protein between both TS and V viruses (**Table 3**). Although the V virus was isolated 4 years later than the TS virus, the phylogenic analysis indicated that each gene segment of both viruses belongs to the CK/BJ-like H9N2 subgroup (Data not shown) and is not derived from other subtypes of influenza A virus.

**Table 2:** Amino acid differences between avian influenza H9N2 TS and V viruses Amino acid differences of surface proteins between avian influenza H9N2 TS and V viruses.

Gene	Position/H9 (H3)	TS	V
HA	3 (–)	A	V
	15 (–)	A	V
	92 (84)	G	R
	107 (99)	M	L
	165 (157)	K	E
	166 (158)	D	N
	171 (163)	I	V
	183 (175)	S	N
	213 (205)	T	A
	224 (216)	V	L
	243 (235)	S	A
	252 (244)	Q	R
	370 (362)	V	T
	469 (461)	M	V
	539 (531)	M	L
NA	22	F	L
	30	A	V
	62–64	–	ITE
	70	S	G
	73	I	L
	81	V	A
	83	E	G
	85	K	R
	141	K	D
	149	T	A
	153	I	T
	170	G	A
	210	M	I
	249	K	R
	296	K	R
	313	K	D
	331	N	R
	332	S	T
	356	N	S
	367	K	E
	368	E	K
	370	L	S
	380	V	T
	384	T	I
	403	W	S
	416	N	S
	432	Q	K

**Table 3:** Amino acid differences of internal gene proteins between avian influenza H9N2 TS and V viruses.

Gene	Amino acid position	TS	V
PB2	60	N	D
	76	T	M
	109	V	I
	188	E	D
	292	I	V
	379	R	K
	555	R	K
	613	V	I
	627	E	K
	649	V	I
PB1	76	D	N
	111	M	I
	157	A	T
	171	T	M
	328	N	K
	368	V	I
	387	K	Q
	566	T	M
	621	Q	K
	744	T	M
PA	14	V	A
	142	R	K
	185	R	K
	254	N	T
	323	V	I
	552	T	N
	683	L	I
	684	G	E
NP	21	N	D
	52	H	Y
	214	R	K
	329	V	I
	417	S	N
	423	A	S
	473	S	N
	496	Y	H
	21	N	D
M2	10	P	H
	16	E	G
	27	V	I
	32	V	I
	85	N	D
NS1	26	G	E
	27	R	L
	59	H	R
	70	E	K
	86	A	V
	95	I	L
	123	I	T
	137	T	I
	179	E	G
	180	I	V
	197	T	N
NS2	11	G	D
	22	R	G
	40	L	I
	63	G	W
	85	H	R

### Amino Acid Substitution at Position 627 in PB2 Protein Changes Virulence of the TS and V Viruses

**Figure 3:**
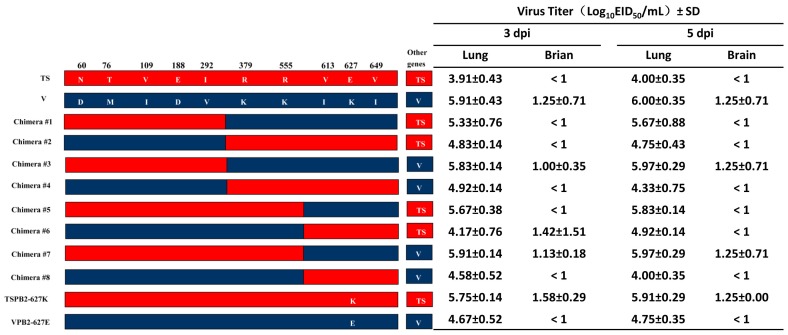
Schematic diagram of chimeric and single amino acid PB2 mutants and virus titers in mouse lungs and brains. Differences of amino acid residues in PB2 between TS and V virus were shown as single-letter amino acid codes with their positions indicated at the top of the diagram. The red and blue bars indicate the amino acid regions originated from TS or V, respectively. Virus titers are presented by log_10_ geometric mean EID_50_/ml ± SEM (The detection limit is 10^1^ EID_50_/ml).

**Figure 4:**
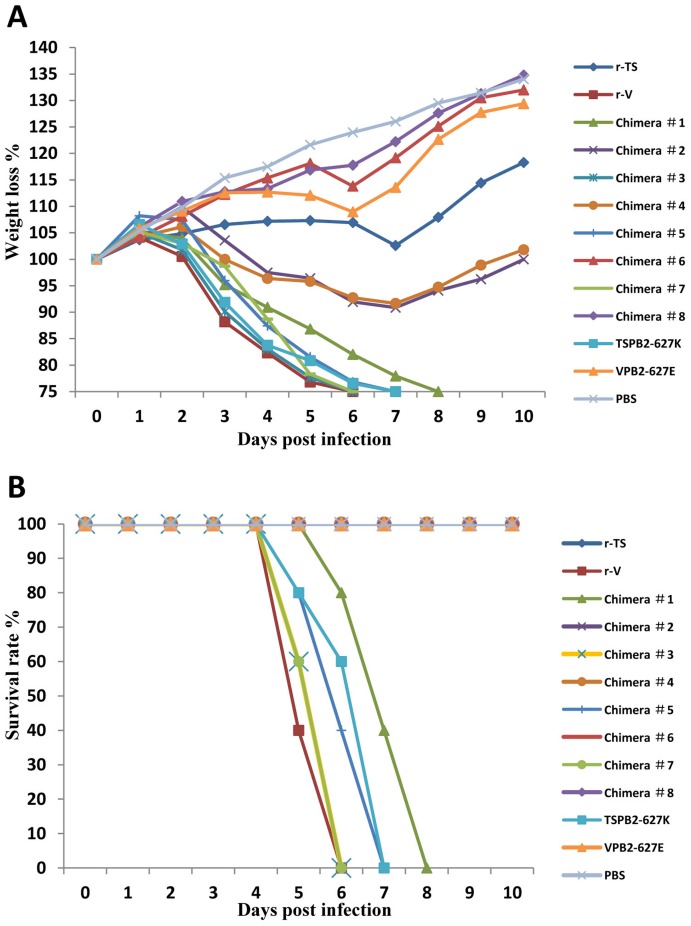
Weight changes and mortality of mice inoculated with parental, chimeric or mutated viruses. A) Weight changes; B) Mortality of mice inoculated with parental, chimeric or mutated viruses.

Sequence analysis showed that there were 10 amino acid differences in the PB2 between the TS and V viruses (**Table 3**); in addition, PB2 played a major role in virus replication, tissue tropism and pathogenicity in the studies of recombinant viruses. To further identify the amino acid(s) in PB2 responsible for the observed difference in viral replication and virulence of TS and V viruses in mice, we generated eight PB2 chimeric viruses and 2 mutated viruses that contain a single substitution at the position of 627 in PB2 (**Figure 3**). Firstly, we generated chimera #1, #2, #3 and #4. The mouse study revealed that Chimera #2 (TS virus possessing the N-terminal portion of the V PB2-1-344aa) and #4 (V virus possessing the C-terminal portion of the TS PB2-345-759aa) induced less than 10% weight loss in infected mice without mortality, whereas the chimera #1 (TS virus possessing the C-terminal portion of the V PB2-345-759aa) and #3 (V virus possessing the N-terminal portion of the TS PB2-1-344aa) caused over 25% weight loss in infected mice with 100% mortality (**Figure 4A and B**). Furthermore, both chimera #1 and #3 replicated to slightly higher virus titers in mouse lungs than the chimera #2 and #4. Only chimera #3 was also detected in the mouse brains (**Figure 3**). These results indicated that the last 5 amino acids at positions 378, 555, 613,627 and 649 located at C-terminal of the PB2 are critical for viral replication and virulence in mice.

To determine which of the 5 amino acids located at the C-terminal of the PB2 is important for virus replication and virulence, we generated chimera #5, #6, #7 and #8 viruses. The results exhibited that Chimera #6 (TS virus possessing the N-terminal portion of the V PB2-1-570aa) and #8 (V virus possessing the C-terminal portion of the TS PB2-571-759aa) did not induce weight loss and clinical signs in infected mice and were similar to the controls (**Figure 4A**). In contrast, the chimera #5 (TS virus possessing the C-terminal portion of the V PB2-571-759aa) and #7 (V virus possessing the N-terminal portion of the TS PB2-1-570aa) caused obvious clinical signs and weight loss with 100% mortality. Moreover, chimera #5 and #7 replicated to higher virus titers in mouse lungs than the chimera #6 and #8. However, only chimera #7 (3 and 5 dpi) and #6 (3 dpi) were also detected in mouse brains (**Figure 3**). These results demonstrated that a maximal of three amino acid positions (V613I, E627K and V649I) at the C-terminus of the PB2 affect virulence of the TS and V viruses in mice.

To determine the importance of amino acid at position 627 in PB2, we generated two mutant viruses TSPB2-627K and VPB2-627E. The mutant VPB2-627E virus encoding E at position 627 of PB2 was not lethal and did not replicate in mouse brains when compared to the parental r-V virus (**Figure 3** **and** **4**). However, the mutant TSPB2-627K encoding K at position 627 of PB2 killed all infected mice and replicated to significantly higher titers in mouse lungs and brains when compared to the parental r-TS virus (**Figure 3** **and** **4**). These results suggest that the amino acid at position 627 of the PB2 is critical for differences in viral replication and virulence between the TS and V viruses in mice.

## Discussion

Several permanent lineages of H9N2 AIVs are established in land-based poultry and have become endemic in Asia [26], [27], [28], [29]. Both V and TS viruses used in this study belong to the CK/BJ-like H9N2 subgroup and are non-pathogenic in chickens although they were isolated from diseased or dead chickens. The diseased or dead chickens in the farms with an outbreak of respiratory disease were most likely caused by multiple pathogens rather than a single H9N2 influenza virus according to the IVPI test. Early H9N2 isolates from chickens replicate poorly or not at all in mice [30]. Previous studies showed that a few H9N2 isolates replicate systemically and are pathogenic for mice without prior adaptation [5], [18], [30], indicating that some H9N2 viruses have gained the ability to cross the species barrier and could replicate in mammalian hosts. A similar situation is also demonstrated with the H9N2 described in this study: the TS virus is nonlethal for mice whereas the V virus is lethal for mice. These findings indicate that some H9N2 isolates have expanded their host range and have adapted to mammalian hosts. Indeed, H9N2 viruses have been isolated from pigs in China and Korea [10], [11], [14], [27], [31] due to avian to pig interspecies transmission; 10 genotype of H9N2 viruses were shown to coexist in pigs in China from 1998 to 2007 [15]. More importantly, some circulating H9N2 viruses obtained typical human-like receptor specificity with amino acid L at position 226 at the receptor binding site [32], resulting in efficient replication in cultured human airway epithelial cells [20]. Interestingly, most of the recent H9N2 isolates from pigs and poultry have 226L in HA [33], indicating that H9N2 viruses are gradually adapting to mammals. In contrast, both the TS and V viruses contain glutamine (Q) at position 226 in the HA, which is a typical avian-like receptor binding marker, binding the SAa2, 3Gal receptors. This result indicates that the HA is not the only determinant which affects replication and pathogenicity of H9N2 AIVs in mice. When compared to virus replication in mice, the V virus replicated a significantly higher titer in mouse lungs than the TS virus and it can enter the central nervous system with a detectable titer. The recombinant viruses such as V-TSPA, -HA, -NA and -NS replicated comparable titers in mouse lungs as the V virus did, but they were not detected from mouse brains. They caused less mortality in mice when compared to the V virus, indicating that replication in brain might enhance viral virulence in mice although virus replication in lungs seems to be the major determinant for the mouse mortality to the infection.

H9N2 viruses have infected humans, leading to mild respiratory disease in people from Hongkong and mainland China during 1999–2003 [12], [16], [17]. The infection of humans with H9N2 viruses resulted in typical human flu-like illness that can be easily associated with seasonal flu viruses and overlooked. H9N2 viruses could have the potential to become a human pandemic strain in addition to the highly pathogenic H5N1 virus, which is considered to be a major pandemic threat [34]; H5N1 viruses cause infection with high mortality in humans and spread from Asia into Africa and Europe due to its association with wild migratory birds [35], [36]. To date, the mechanism of adaptation and interspecies transmission of H9N2 viruses from birds to mammals remains poorly understood. Our present study using two H9N2 field chicken isolates showed the adaptation and pathogenicity of H9N2 influenza viruses in mice is largely attributed to the PB2 gene. Previous studies have shown that PB2 627K in human influenza viruses and highly pathogenic H5N1 AIVs plays an important role in the host range and replication in mammalian hosts [22], [23], [37]. Adaptation of H9N2 virus to quail and chickens facilitates this strain with expanded host range, i.e., it was shown that an H9N2 virus replicates more efficiently in mice and the mouse-adapted virus has the mammalian signature 627K in PB2 after adaptation to land- based poultry [3]. A serially passaged chicken H9N2 virus in mouse lungs also contains PB2 627K [24]. All these results suggest that PB2 627K is very important for virulence and mouse-adaptation of the H9N2 virus. To our knowledge, to date only one H9N2 field strain was found to have PB2 627K prior to this study, which was isolated from the ostrich in South Africa (A/ostrich/South Africa/9508103/95, GenBank accession No. AF508640). Herein, we showed that a chicken field H9N2 isolate already contains PB2 627K that is crucial for pathogencity and tissue tropism in mice. This finding suggests that H9N2 viruses started to adapt to the mammalian hosts not only on the HA but also the PB2 gene. Surprisingly, the H9N2 virus with PB2 627K is first isolated from poultry rather than from the mammalian hosts such as swine or humans. This scenario is somehow different with the H5N1 virus where the PB2 627K is repeatedly reported in human cases [38] and other mammalian species such as tigers [39], dogs [40] and cats [41]. Whether the H9N2 virus will enhance replication and virulence in humans and swine with the acquisition of PB2 627K needs to be investigated.

All chimeric V viruses containing V PB2 C-terminal with 627K are able to replicate in mouse brains. In the TS background, V PB2 C-terminal 627K chimeric viruses did not replicate in mouse brains although the recombinant virus TS-VPB2 does, indicating that other amino acids except 627K also play a role in viral replication in mouse brains. The chimera #6 (TS virus possessing the N-terminal portion of the V PB2-570aa) replicated in mouse brains on 3 dpi, whereas the chimera #2 (TS virus possessing the N-terminal portion of the V PB2-344aa) did not. This result indicates that amino acids 379K and 555K are critical for replication of TS chimeric viruses in mouse brains since there are only 2 amino acid differences between chimera #2 and #6.

Besides PB2, other genes are also associated with pathogenicity of influenza A virus. Several genes from the TS virus attenuated the V virus, especially the PB1, NP or M genes. All of these recombinant viruses caused less mortality although they still caused disease in mice, indicating that these genes are important for the virulence of the V virus. Vice versa, the PA or NA gene from the V virus enhanced pathogenicity of the TS virus when compared to the parental r-TS virus. Furthermore, the NP gene affected viral replication in mouse lungs in both TS and V genetic background although not as prominent as the PB2 did. All these results suggest that the pathogenicity of influenza viruses is a polygenic trait. Which domain or amino acid (s) within the above described genes contributes to pathogenicity in mice needs to be investigated in future studies.

The polymerase subunit PB1, PB2, PA along with NP and viral RNA form the ribonucleoprotein which is responsible for viral replication and transcription. Numerous studies have shown that the amino acid at position 627 in PB2 affects polymerase activity and replication efficiency [42]. Our findings support this conclusion since the H9N2 virus with PB2 627K produced higher virus titers in lungs when compared to the virus with PB2 627E in mice. However, other residues of the PB2 also seem to have impact on viral replication and tissue tropism of H9N2 viruses in mice; how and which specific residues of the PB2 need to be determined in future studies.

Taken together, we showed a mammalian adapted signature PB2 627K in an H9N2 field isolate and demonstrated that PB2 627K plays an important role for the virulence and tissue tropism of H9N2 AIVs in mice. This study supports the previous finding that PB2 627K is an important determinant of host range and virulence of influenza A viruses.

## References

[1] Homme PJ, Easterday BC (1970). Avian influenza virus infections. I. Characteristics of influenza A-turkey-Wisconsin-1966 virus.. Avian Dis.

[2] Kawaoka Y, Chambers TM, Sladen WL, Webster RG (1988). Is the gene pool of influenza viruses in shorebirds and gulls different from that in wild ducks?. Virology.

[3] Hossain MJ, Hickman D, Perez DR (2008). Evidence of expanded host range and mammalian-associated genetic changes in a duck H9N2 influenza virus following adaptation in quail and chickens.. PLoS One.

[4] Aamir UB, Wernery U, Ilyushina N, Webster RG (2007). Characterization of avian H9N2 influenza viruses from United Arab Emirates 2000 to 2003.. Virology.

[5] Guo YJ, Krauss S, Senne DA, Mo IP, Lo KS (2000). Characterization of the pathogenicity of members of the newly established H9N2 influenza virus lineages in Asia.. Virology.

[6] Kim JA, Cho SH, Kim HS, Seo SH (2006). H9N2 influenza viruses isolated from poultry in Korean live bird markets continuously evolve and cause the severe clinical signs in layers.. Vet Microbiol.

[7] Naeem K, Ullah A, Manvell RJ, Alexander DJ (1999). Avian influenza A subtype H9N2 in poultry in Pakistan.. Vet Rec.

[8] Alexander DJ (2000). A review of avian influenza in different bird species.. Vet Microbiol.

[9] Nili H, Asasi K (2003). Avian influenza (H9N2) outbreak in Iran.. Avian Dis.

[10] Cong YL, Pu J, Liu QF, Wang S, Zhang GZ (2007). Antigenic and genetic characterization of H9N2 swine influenza viruses in China.. J Gen Virol.

[11] Cong YL, Wang CF, Yan CM, Peng JS, Jiang ZL (2008). Swine infection with H9N2 influenza viruses in China in 2004.. Virus Genes.

[12] Peiris JS, Guan Y, Markwell D, Ghose P, Webster RG (2001). Cocirculation of avian H9N2 and contemporary “human” H3N2 influenza A viruses in pigs in southeastern China: potential for genetic reassortment?. J Virol.

[13] Xu C, Fan W, Wei R, Zhao H (2004). Isolation and identification of swine influenza recombinant A/Swine/Shandong/1/2003(H9N2) virus.. Microbes Infect.

[14] Yu H, Hua RH, Wei TC, Zhou YJ, Tian ZJ (2008). Isolation and genetic characterization of avian origin H9N2 influenza viruses from pigs in China.. Vet Microbiol.

[15] Yu H, Zhou YJ, Li GX, Ma JH, Yan LP (2010). Genetic diversity of H9N2 influenza viruses from pigs in China: a potential threat to human health?. Vet Microbiol.

[16] Butt KM, Smith GJ, Chen H, Zhang LJ, Leung YH (2005). Human infection with an avian H9N2 influenza A virus in Hong Kong in 2003.. J Clin Microbiol.

[17] Lin YP, Shaw M, Gregory V, Cameron K, Lim W (2000). Avian-to-human transmission of H9N2 subtype influenza A viruses: relationship between H9N2 and H5N1 human isolates.. Proc Natl Acad Sci U S A.

[18] Choi YK, Ozaki H, Webby RJ, Webster RG, Peiris JS (2004). Continuing evolution of H9N2 influenza viruses in Southeastern China.. J Virol.

[19] Matrosovich MN, Gambaryan AS, Teneberg S, Piskarev VE, Yamnikova SS (1997). Avian influenza A viruses differ from human viruses by recognition of sialyloligosaccharides and gangliosides and by a higher conservation of the HA receptor-binding site.. Virology.

[20] Wan H, Perez DR (2007). Amino acid 226 in the hemagglutinin of H9N2 influenza viruses determines cell tropism and replication in human airway epithelial cells.. J Virol.

[21] Hatta M, Gao P, Halfmann P, Kawaoka Y (2001). Molecular basis for high virulence of Hong Kong H5N1 influenza A viruses.. Science.

[22] Li Z, Chen H, Jiao P, Deng G, Tian G (2005). Molecular basis of replication of duck H5N1 influenza viruses in a mammalian mouse model.. J Virol.

[23] Subbarao EK, London W, Murphy BR (1993). A single amino acid in the PB2 gene of influenza A virus is a determinant of host range.. J Virol.

[24] Wu R, Zhang H, Yang K, Liang W, Xiong Z (2009). Multiple amino acid substitutions are involved in the adaptation of H9N2 avian influenza virus to mice.. Vet Microbiol.

[25] Chen GW, Chang SC, Mok CK, Lo YL, Kung YN (2006). Genomic signatures of human versus avian influenza A viruses.. Emerg Infect Dis.

[26] Guan Y, Shortridge KF, Krauss S, Chin PS, Dyrting KC (2000). H9N2 influenza viruses possessing H5N1-like internal genomes continue to circulate in poultry in southeastern China.. J Virol.

[27] Dong G, Luo J, Zhang H, Wang C, Duan M (2010). Phylogenetic diversity and genotypical complexity of H9N2 influenza A viruses revealed by genomic sequence analysis.. PLoS One.

[28] Cameron KR, Gregory V, Banks J, Brown IH, Alexander DJ (2000). H9N2 subtype influenza A viruses in poultry in pakistan are closely related to the H9N2 viruses responsible for human infection in Hong Kong.. Virology.

[29] Lee YJ, Shin JY, Song MS, Lee YM, Choi JG (2007). Continuing evolution of H9 influenza viruses in Korean poultry.. Virology.

[30] Li C, Yu K, Tian G, Yu D, Liu L (2005). Evolution of H9N2 influenza viruses from domestic poultry in Mainland China.. Virology.

[31] Shi WF, Gibbs MJ, Zhang YZ, Zhang Z, Zhao XM (2008). Genetic analysis of four porcine avian influenza viruses isolated from Shandong, China.. Arch Virol.

[32] Matrosovich MN, Krauss S, Webster RG (2001). H9N2 influenza A viruses from poultry in Asia have human virus-like receptor specificity.. Virology.

[33] Sun Y, Pu J, Jiang Z, Guan T, Xia Y (2010). Genotypic evolution and antigenic drift of H9N2 influenza viruses in China from 1994 to 2008.. Vet Microbiol.

[34] Alexander PE, De P, Rave S (2009). Is H9N2 avian influenza virus a pandemic potential?. Can J Infect Dis Med Microbiol.

[35] Alexander DJ (2007). Summary of avian influenza activity in Europe, Asia, Africa, and Australasia, 2002–2006.. Avian Dis.

[36] Alexander DJ, Brown IH (2009). History of highly pathogenic avian influenza.. Rev Sci Tech.

[37] Gabriel G, Dauber B, Wolff T, Planz O, Klenk HD (2005). The viral polymerase mediates adaptation of an avian influenza virus to a mammalian host.. Proc Natl Acad Sci U S A.

[38] Le QM, Sakai-Tagawa Y, Ozawa M, Ito M, Kawaoka Y (2009). Selection of H5N1 influenza virus PB2 during replication in humans.. J Virol.

[39] Amonsin A, Payungporn S, Theamboonlers A, Thanawongnuwech R, Suradhat S (2006). Genetic characterization of H5N1 influenza A viruses isolated from zoo tigers in Thailand.. Virology.

[40] Songserm T, Amonsin A, Jam-on R, Sae-Heng N, Pariyothorn N (2006). Fatal avian influenza A H5N1 in a dog.. Emerg Infect Dis.

[41] Songserm T, Amonsin A, Jam-on R, Sae-Heng N, Meemak N (2006). Avian influenza H5N1 in naturally infected domestic cat.. Emerg Infect Dis.

[42] Shinya K, Hamm S, Hatta M, Ito H, Ito T (2004). PB2 amino acid at position 627 affects replicative efficiency, but not cell tropism, of Hong Kong H5N1 influenza A viruses in mice.. Virology.

